# Genome sequencing of severe acute respiratory syndrome COVID-19 EF.1 variant strain obtained from a Moroccan patient

**DOI:** 10.1128/mra.01255-23

**Published:** 2024-03-19

**Authors:** Lahcen Ouchari, Mouhssine Hemlali, Hamza Ghammaz, Taha Chouati, Marouane Melloul, Sanaâ Alaoui Amine, Safaa Rhoulam, Hicham Elannaz, Nadia Touil, Khalid Ennibi, Elmostafa El Fahime

**Affiliations:** 1 Molecular Biology and Functional Genomics Platform, National Center for Scientific and Technical Research (CNRST), Rabat, Morocco; 2 Microbiology and Molecular Biology Team, Plant and Microbial Biotechnology, Biodiversity and Environment Center, Faculty of Sciences, Mohammed V University in Rabat, Rabat, Morocco; 3 Neuroscience and Neurogenetics Research Team, Faculty of Medicine and Pharmacy, University Mohammed V, Rabat, Morocco; 4 Cell Culture Unit, Center of Virology, Infectious, and Tropical Diseases, Med V Military Hospital, Rabat, Morocco; 5 Genomic Center for Human Pathologies (GENOPATH), Faculty of Medicine and Pharmacy, University Mohammed V, Rabat, Morocco; DOE Joint Genome Institute, Berkeley, California, USA

**Keywords:** COVID-19, genome sequencing, subvariant, immune evasion

## Abstract

Here, we report the identification and coding-complete genome sequence of a severe acute respiratory syndrome COVID-19 (SARS-CoV-2) strain obtained from a Moroccan patient. The detected strain EF.1 belongs to the BQ1.1 subvariant of the BA.5 Omicron variant.

## ANNOUNCEMENT

Severe acute respiratory syndrome COVID-19 (SARS-CoV-2 virus; genus: *Betacoronavirus*; family: *Coronaviridae*), responsible for COVID-19 pandemic, remains a substantial concern due to the mutations in variants that could cause a resurgence (1, 2). Two prominent variants, BQ.1 and BQ.1.1, were initially detected in Nigeria in July 2022 and quickly considered variants of interest (VOIs) (3) due to their increasing complexity of spike mutations which enhance their ability to evade neutralizing antibodies, even in individuals who have received a bivalent mRNA booster or have previously encountered breakthrough Omicron infections (4).

In the evolution monitoring of the SARS-CoV-2 virus, a sample was obtained from an asymptomatic patient from Rabat (Morocco) on 2023-05-01. The viral RNA was extracted using a MagPurix viral RNA extraction kit (Zinexts Life Science, China). Reverse transcription (SARS-CoV-2-specific primers) was performed using SuperScript VILO cDNA synthesis kit (Invitrogen, Thermo Fisher Scientific, USA) and a library was prepared according to the instruction of Ion AmpliSeq SARS-CoV-2 research panel (Thermo Fisher, USA). Template preparation and chip loading were performed using the Ion Chef system, and sequenced by the Ion S5 sequencer (Thermo-fisher Scientific, USA). In all, 1,927,688 reads (Average length: 188.38 bp; GC content: 37.83%) were obtained and aligned to the Wuhan-Hu-1 reference sequence (GenBank accession number NC_045512.2) using Unipro UGENE V.45 to generate a consensus sequence. Default parameters were used.

The obtained genome (hCoV-19/Morocco/117/2023) presented a length of 29,841 bp, which covered 99.6288% of the reference genome (average coverage depth: 11916.9×). The missing portions were the 5′ and 3′ UTRs. Sequence quality check was performed using Nextclade web tool v2.14.0 (https://clades.nextstrain.org) and mutation identification was carried out using the web application Cov-GLUE (http://cov-glue.cvr.gla.ac.uk/). Lineage and sublineage were obtained using the Pangolin web application v4.0 (https://pangolin.cog-uk.io/).

**Fig 1:**
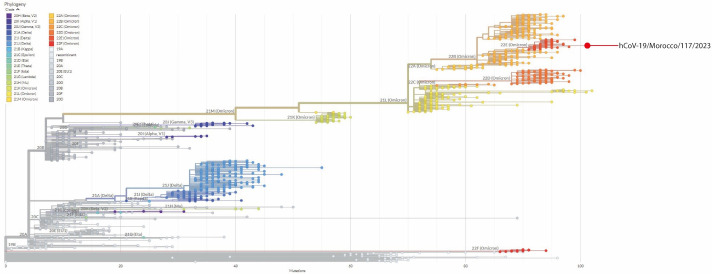
Phylogenetic tree displaying the sequenced SARS-CoV-2 strain (with reference to the global GISAID data set), according to the clades assigned to the virus in the phylogenetic analysis using the Augur toolkit (with default settings) run by the Nextstrain server. The red dot marking the location of the strain hCoV-19/Morocco/117/2023 is part of the 22E Omicron clade. However, it shows a high number of mutations compared to the closely related strains.

According to the Pangolin web application, the sample belongs to the sublineage BQ1.1 of the Omicron variant. According to Nextclade, on the other hand, it was part of the clade 22E. The viral genome presented a total of 74 mutations (Table 1), and their locations in the global diversity of SARS-CoV-2 are illustrated in Fig. 1. Notably, 27 of the total mutations were located at the level of the S Protein gene, and contained well-known mutations such as D614G, which is also present in most variants including alpha and delta (5). Other substitutions observed were characteristic of BQ1.1, such as E484A (6). This latter mutation is extensively investigated for its impact on the binding affinity of the spike to the ACE2 receptor, whose increase helps viral entry into host cells (2, 7). Moreover, it was linked to a reduction in the neutralization potency of antibodies, suggesting a potential immune evasion mechanism (2).

**TABLE 1:** Amino acids changes of SARS-CoV-2 strain hCoV-19/Morocco/117/2023 in comparison to the reference strain (GenBank accession number NC_045512.2)

Protein	Amino acids changes
*NSP1*	S135R
*NSP2*	Q376K
*NSP3*	T24I, G489S
*NSP4*	L264F, T327I, T492I
*NSP5*	P132H
*NSP6*	F108del, G107del, L260F, S106del
*NSP12*	P323L, Y273H
*NSP13*	M233I, N268S, R392C
*NSP14*	I42V
*NSP15*	T112I
*Spike*	A27S, D405N, D614G, D796Y, E484A, F486V, G142D, G339D, H69del, H655Y, K417N, K444T, L24del, L452R, N440K, N460K, N679K, N764K, N969K, P25del, P26del, P681H, Q954H, S371F, S373P, S375F, S477N, T19I, T376A, T478K, V70del, V213G, Y144del
*NS3*	A72T, T223I
*E*	T9I, V62F
*M*	A63T, D3N, Q19E
*N*	E31del, E136D, G204R, P13L, R32del, R203K, S33del, S413R

Other interesting mutations were observed, such as S477N of the Receptor-Binding Domain (RBD) of the Spike protein (8), N679K and P681H between the S1 and S2 subunits (9, 10), as well as G142D in the NTD region (a crucial binding site for antibody 4A8) (11, 12).

The subvariant BQ1.1 and its emergence is a significant challenge in the fight against COVID-19. It shows an ability to evade immune responses and alter the virus biology, which raises concerns about vaccine effectiveness and current control measures. This only helps emphasize the importance of continued research and a better understanding of the prevalence, transmissibility, and clinical impact of the Omicron EF.1 variant.

## Data Availability

The SARS-CoV-2 genome sequence was submitted to the GISAID database under the identifier EPI_ISL_17685328 and to NCBI GenBank under the accession number OR002180. Raw reads from next-generation sequencing (NGS) are available in the SRA under the run accession SRR24761726 and in BioProject under the accession number PRJNA977611.
